# Estimating the Burden of Acute Gastrointestinal Illness: A Pilot Study of the Prevalence and Underreporting in Saint Lucia, Eastern Caribbean

**Published:** 2013-12

**Authors:** Owen O. Gabriel, Alina Jaime, Martin Mckensie, Ava Auguste, Enrique Pérez, Lisa Indar

**Affiliations:** ^1^Ezra Long Laboratory, Victoria Hospital, Ministry of Health, Saint Lucia; ^2^Epidemiology Unit, Ministry of Health, Saint Lucia; ^3^Pan American Health Organization, Panama; ^4^Caribbean Epidemiology Centre (CAREC/PAHO/WHO), Trinidad and Tobago

**Keywords:** Acute gastroenteritis, Burden of illness, Diarrhoea, Foodborne pathogens, Underreporting, Saint Lucia

## Abstract

Saint Lucia was the first country to conduct a burden of illness study in the Caribbean to determine the community prevalence and underreporting of acute gastroenteritis (AGE). A retrospective cross-sectional population survey on AGE-related illness was administered to a random sample of residents of Saint Lucia in 20 April–16 May 2008 and 6-13 December 2009 to capture the high- and low-AGE season respectively. Of the selected 1,150 individuals, 1,006 were administered the survey through face-to-face interviews (response rate 87.4%). The overall monthly prevalence of AGE was 3.9%. The yearly incidence rate was 0.52 episodes/person-year. The age-adjusted monthly prevalence was 4.6%. The highest monthly prevalence of AGE was among children aged <5 years (7.5%) and the lowest in persons aged 45-64 years (2.6%). The average number of days an individual suffered from diarrhoea was 3.8 days [range 1-21 day(s)]. Of the reported AGE cases, only seven (18%) sought medical care; however, 83% stayed at home due to the illness [(range 1-16 day(s), mean 2.5]; and 26% required other individuals to take care of them. The estimated underreporting of syndromic AGE and laboratory-confirmed foodborne disease pathogens was 81% and 99% respectively during the study period. The economic cost for treating syndromic AGE was estimated at US$ 3,892.837 per annum. This was a pilot study on the burden of illness (BOI) in the Caribbean. The results of the study should be interpreted within the limitations and challenges of this study. Lessons learnt were used for improving the implementation procedures of other BOI studies in the Caribbean.

## INTRODUCTION

Foodborne diseases (FBDs) are an important cause of morbidity, mortality, and public-health concern worldwide (1,2). Acute gastroenteritis (AGE), the key syndrome relating to food- and waterborne illnesses, is increasing globally. Monthly prevalence estimates of AGE in developed countries range between 4.5 and 11% (3). Although typically mild and self-limiting, AGE imposes a substantial economic burden on the population and healthcare system (3-9). Diarrhoea is a major cause of morbidity and mortality among under-5 children in developing countries. It is estimated that there are 3.2 episodes of diarrhoea/child/year and 4.9 deaths/1,000 children/year due to diarrhoeal illness (7,10,11). Precise information on the burden of illness (BOI) is needed to facilitate control efforts for foodborne diseases (FBDs). As part of a strategy to reducing foodborne diseases globally, the World Health Organization (WHO), through the Global Burden of Disease initiative, has developed a rigorous approach for BOI estimations (12,13)

Saint Lucia is a developing nation of Eastern Caribbean. The total mid-year population was estimated at 156,635 in 2006 (14). The island is divided into 8 health regions. Majority of the population inhabits the coastal areas and less mountainous regions of the north and south of the island, with approximately 41.0% of the population living in the northern district of Castries. The Ministry of Health (MOH) is the sole provider of health services in the public sector. Health services delivery is provided through a network of primary and secondary care services. Primary healthcare services are provided at 33 health centres, a polyclinic, and 2 district hospitals. Victoria Hospital is the main hospital and is located in the City of Castries and is managed by the Ministry of Health. St. Jude's Hospital is located in the south of the island and is a quasi-government institution. Approximately 95.0% of urban households and 88.0% of rural households have access to safe/potable water (15). Tourism is the principal engine of economic growth in Saint Lucia and accounted for 13.6% of real GDP in 2006 (15,16).

In Saint Lucia, the epidemiology of food- and waterborne diseases at the community level is unknown. Little information is available on the magnitude and burden of these illnesses and on the key pathogens responsible for food- and waterborne infections, thereby limiting appropriate prevention measures. Currently, most people affected by AGE are not captured by the traditional national surveillance systems, leading to significant underreporting of this syndrome. Weekly tallies of syndromic AGE cases are generated in the community, hospitals, laboratories, polyclinics, three sentinel sites (Victoria Hospital, St. Jude's Hospital, and Gros Islet Polyclinic), and private primary-care physicians. Data are collected by the Ministry of Health. These data indicated a total of 3,893 reported cases of AGE in 2006 and 1,042 cases in 2007. Laboratory data, however, indicated that, in 2006, approximately 329 stool specimens were submitted, and only 9 cases of *Salmonella* and 16 cases of *Shigella* were identified while, in 2007, approximately 225 stool specimens were submitted, and 13 cases of *Salmonella* and 8 cases of *Shigella* were identified (17). These data suggest a potential high burden of gastroenteritis and foodborne diseases, considering the small population-size and the low number of stool samples submitted and tested. Conducting BOI study in St. Lucia is, therefore, imperative to determine the true burden and the economic impact of acute gastroenteritis, foodborne diseases, and the key causal pathogens to guide appropriate prevention and control strategies and for 2006 allocation of limited resources intended for the health sector.

Consequently, St. Lucia joined the Caribbean Burden of Illness Study led by CAREC in collaboration with Pan American Health Organization (PAHO) in 2008. St. Lucia was the first country in this study to execute a BOI study in the Caribbean. The objective of this pilot study, conducted by the Ministry of Health of Saint Lucia in collaboration with CAREC and PAHO, was to determine the prevalence, burden, and underreporting of common pathogens causing acute gastrointestinal illness (AGI).

## MATERIALS AND METHODS

Saint Lucia was selected as the pilot site for this study as it was the first in the Caribbean region to implement enhanced surveillance consisting of a an integrated laboratory, hospital, and community surveillance system involving all relevant health sectors, including veterinary, environmental, and ports. The study consisted of two survey components—a population-based and a laboratory-based study. Formal ethical approval was obtained for this study from the Ethics Committee of the Ministry of Health (MOH) of Saint Lucia.

### Population survey component

A cross-sectional face-to-face survey of randomly-selected residents was administered during 20 April–16 May 2008 (Phase 1: high-AGE season) and 6-13 December 2009 (Phase 2: low-AGE season). The high and low seasons for AGE were designated based on a 5-year syndromic AGE trends over the period 2002-2006. Trained interviewers conducted the surveys via face-to-face interviews. In the first phase, 20 trained nurse supervisors working in primary healthcare in communities throughout the island conducted face-to-face interviews during the routine working hours from Monday through Friday at 8:30 am to 4:30 pm. Third-year nursing students were employed to verify information obtained in sets of questionnaire when these were incomplete or clarification was required. However, in the second phase, a survey coordinator was designated, and 30 trained enumerators were contracted from the Statistics Unit. They conducted interviews throughout the entire week, including weekends and whatever times of the day and night the selected respondents were available.

### Sample-size

Using the population census, sample-size calculations were based on an estimated AGE prevalence of 40%, a response rate of 80%, an allowable error of 2%, and a 95% confidence interval. The total sample-size of 1,150 was calculated using Epi Info. The target sample-size in Phase 1 (high-AGE season) and in Phase 2 (low-AGE season) was 650 and 500 respectively.

### Selection of households and respondents

Houses were first randomly selected using a sampling frame provided by the Saint Lucia Central Statistics Office. Once a house had been selected, the individual in the house was selected with the next birthday falling before the day of the interview. If these individuals were aged less than 12 years, their parents or guardians were asked to respond on their behalf. If the individuals were between 12 and 17 years of age, permission to participate in the study was obtained from their parents or guardians. If a selected individual was unavailable, the surveyor was required to schedule a follow-up visit to the home. If after 3 attempts, the selected individual was unavailable, the surveyor would move on to the next closest residence to select another individual. Individuals were excluded from the survey if they were less than one year of age or less than 18 years of age without parental consent, unwilling or unable to participate in the study, not physically present in the country at the time of the survey, and were prisoners or mentally disabled.

### Collection of data

A standard written questionnaire designed by the Caribbean BOI study team was used for administering the survey. This was modified for selected risk factor variables and tailored to suit country specificity of nomenclature of particular foods (18) and clarity for accommodating the Creole Patois language largely spoken in the rural areas in consultation with the Community Health Nursing Division, National Epidemiology Unit and Laboratory of Saint Lucia. Each questionnaire was numbered and used in identifying the respondent in the database. Respondents were asked if they experienced symptoms of diarrhoea. A case of AGE was defined as anyone who experienced diarrhoea in the 28 days prior to the interview, resulting in 3 or more loose stools in a 24-hour period and who did not declare to have a chronic condition causing diarrhoea (12). Individuals with an unknown number of loose stools in the 24 hours, had diarrhoea caused by medications, laxatives or alcohol, were not considered to be cases. Additional questions were asked about sociodemographic factors, secondary symptoms, hygiene and healthcare-seeking behaviour, number of missed days at school or workplace, and whether hospitalization was required.

### Data-entry and statistical analysis

Data were entered manually in EpiData (version 3.1) and validated following duplicate data-entry. Analysis was performed using the Epi Info (version 3.5.1) software (Centers for Disease Control and Prevention, USA) as well as Microsoft Excel 2007 (Microsoft Corporation, One Microsoft Way, Redmond, WA 98052-6399). Univariate analysis was performed on the overall dataset. Prevalence and incidence rates were calculated using the formulae shown in Appendix 1.

### Estimation of underreporting of AGE

The burden of AGE and the level of underreporting were calculated using syndromic data and data from the population and laboratory surveys. The BOI pyramid was defined using the percentage of self-reported cases who sought medical care to estimate underreporting relative to syndromic AGE. The percentages of AGE cases who sought medical care, submitted stool samples, samples tested positive for foodborne pathogens, and reported to the national surveillance, were used in estimating underreporting relative to laboratory-confirmed FBD pathogen.

### Estimation of economic burden of AGE

The potential economic burden of AGE-related illness was estimated by multiplying the estimated episodes in the population per year by the cost of accessing healthcare, treatment for AGE, and average loss of workdays (Appendix 2).

### Laboratory survey component

Data were collected from the Ezra Long Laboratory during the 12-month period of March 2008 to March 2009 on the number of stool specimens submitted by persons with acute gastrointestinal illness (AGI) during that period, the proportion of these specimens which were tested, and the number of specimens testing positive. Ezra Long Laboratory is the National laboratory where all samples of AGI were processed during the period. Specimens were collected and delivered to the laboratory within 24 hours by nurse supervisors from health centres, polyclinics, private physicians’ offices, and district hospitals across the island. Enhanced testing was performed according to the standard methods (BAM 2008) for *Salmonella, Shigella, Campylobacter, Staphylococcus aureus, Escherichia coli (STEC),* rotavirus, and norovirus. This information and data from the syndromic surveillance were used in estimating the magnitude of underreporting of confirmed cases of these pathogens to the national surveillance system.

## RESULTS

### Response rate and characteristics of respondents

In total, 1,150 persons were contacted to participate, of whom 1,006 responded to the surveys—515 in Phase 1 (April–May 2008) and 491 in Phase 2 (December 2008), with an overall response rate of 87.5%. Table 1 summarizes methodology, sample-size, and response rate for the population-based survey.

**Figure 1.**
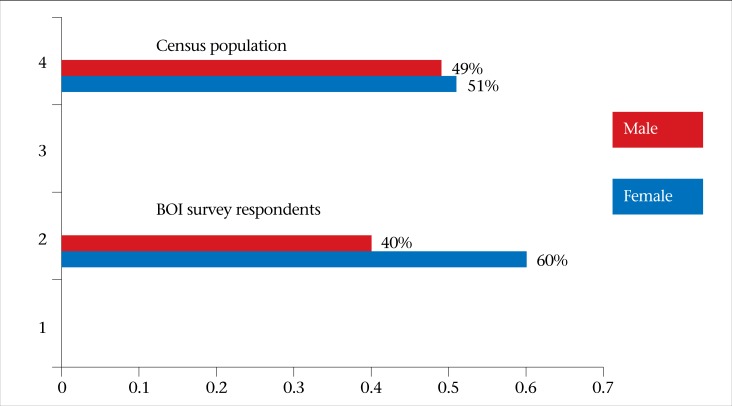
Gender distribution in the Burden of Illness (BOI) Study and 2009 Census population in Saint Lucia

Comparison of the survey respondents with those in the population survey indicates that, overall, respondents were older than the 2009 Census population in St. Lucia, had a higher income, had a higher educational level, and were more likely to be female (Figure 1). The demographic distribution of residents and respondents are outlined in Table 2.

### Magnitude of illness

Of the 1,006 individual surveys completed, 63 (6.3%) respondents reported that they had diarrhoea in the 28 days prior to their interview. Of those 63 individuals, 24 were excluded from AGE cases since some reported their diarrhoea was due to a long-lasting condition (9 cases), some reported having only up to 2 stools in a 24-hour period (9 cases), and some did not indicate the maximum number of stools in a 24-hour period (6 cases). Therefore, 39 of the 1,006 (3.9%) respondents met the case definition for AGE (19 cases in Phase 1 and 20 cases in Phase 2), resulting in an overall monthly prevalence of 3.9% (95% CI 2.8-5.3) (Table 2). The age-adjusted overall monthly prevalence was 4.5%. The yearly incidence rate was 0.52 episodes per person-year.

**Table 1.** Methodology, sample-size, and response rate of respondents in the population-based survey of acute gastrointestinal illness (AGI) in Saint Lucia

	High-AGI season	Low-AGI season	Overall
Study period	20 April–16 May 2008	6-13 December 2008	-
Sample-size (N)	650	500	1,150
Response rate (%)	515 (79%)	491 (98%)	1,006 (87%)
Study area		Saint Lucia	
Population in study area	-	156,635	-
Survey design	-	Retrospective cross-sectional population survey	-
Sampling frame	-	Stratified by regions based on population-size/proportion	-
Household selection	-	Random selection of houses in each enumeration district	-
Individuals selection		Persons with the next birthday		
Type of interview	Face-to-face	Face-to-face	-
Timing of interviews	Evening and night	Daytime, evening, and night	-
Interviewers	Nurses only	Student nurses and enumerators	-
Contact attempts	Up to 3	Up to 3	-

Individual prevalence rates for Phase 1 and Phase 2 of the survey were not statistically different (p=0.75). Overall, the monthly prevalence of AGE was higher among females than males, which was not statistically different (p<0.005) (Table 3a and 3b). The highest monthly prevalence of AGE was among children aged <5 years and persons aged 15-24 years (Table 4a and 4b). The lowest reported prevalence of AGE was among persons aged 45-64 years and 65 years and over (Table 2). The prevalence of AGE by age-group, gender, education of male and female household heads, cultural group, and monthly household income are outlined in Table 2 through 4. In univariate analysis, none of these sociodemographic factors was found to have a significant association with being a case of AGE.

### Symptoms and severity

Additional symptoms experienced by cases are outlined in Table 5. Of the 39 AGE cases, 23 (58.9%) said that they experienced abdominal pain, and nearly a quarter of the cases experienced vomiting (n=9), headache (n=9), or nausea (n=8). There was no significant difference in any of these secondary symptoms between Phase 1 and Phase 2. The maximum number of stools per 24 hours ranged from 3 to 24, with a mean of 4.8 and a median of 4. The average number of days an individual suffered from AGE was 3.8, with a range of 1-21 day(s) and a median of 2 days.

### Medical care and treatment

Of  the 39 cases, 7 (18%) sought medical care for their illness. One (2.5%) attended an outpatient clinic at a private hospital, two (5%) attended an outpatient clinic at a public hospital, three (7.5%) attended a health centre, and one went to a pharmacy. No one visited a traditional healer, an alternative healthcare practitioner, or a private doctor's clinic. None of the cases reported having been hospitalized. Only one case had a stool specimen requested for. Seven cases (18%) reported having medication prescribed. These were the same 7 persons who sought medical care. Three were prescribed antibiotics, two of whom completed the course of antibiotics and one of whom did not, for an unknown reason. Three individuals were prescribed oral rehydration solution. One individual was prescribed vitamins and worm treatment medication. Additionally, nine cases took non-prescribed medications for their illnesses. Of these individuals, four took ‘unknown bush medicine’, three took ‘Pepto-Bismol’, and two took pain killers.

Of the 39 cases, 30 (80%) had to spend time at home due to their illness. This high proportion speaks of a significant economic burden, in addition to the direct costs involved. The range of days spent at home due to illness was 1-14 day(s), with an average of 2.5 days and a median of 1 day. Ten cases required other individuals to look after them while ill. The range of days taking care of a case was 1-14, with an average of 3.2 days and a median of 2 days.

### Risk factors and habits

Individuals were asked to identify what they believed to have caused their illness. Twelve of the 39 cases (31%) believed they became ill from something they consumed, nine (23%) believed that water was the cause, two (5.1%) believed contact with another sick person was the cause, three (7.6%) believed their illness to be caused by something non-infectious, and 13 were unsure of the cause. Among the 12 who believed their illness was caused by something they consumed, the identified items were: cold soup, fish, leftover chicken, mangoes, meat patties, and octopus. The three who believed their illness was not due to an infectious cause mentioned a purge, eating late, and not eating.

### Household-size

Respondents were asked about the number of individuals living in the household; this ranged from 1 to 17 person(s), with an average of 3.6 and a median of 3 persons in the household. Households of non-cases had a median of 3 persons while households of cases had a median of 4 persons. This difference had a borderline significance (p=0.06). Furthermore, coming from a household with 5 or more persons resulted in a 2.1 times increase in the odds of being a case of AGE (p=0.03). Additionally, 7 of the 39 cases (18%) reported that another individual was ill with diarrhoea in their home at the same time of their illness. Five reported one additional individual to be ill, one reported 3 others ill, and one reported 5 others ill.

**Table 2.** Demographic characteristics of residents and survey respondents and monthly prevalence of acute self-reported gastrointestinal illness per category, Saint Lucia

Variable	Residents (N=156,635)^1^	Respondents (n=1,006)	Monthly prevalence	95% Confidence interval	p value
Sex					
Male	77,664	405	3.2	1.7-5.5	0.42
Female	78,971	601	4.4	2.9-6.3	
Age (completed years)					
<1	14,335	8	12.5	0.3-52.7	0.02
1-4	-	40	7.5	1.6-20.4	
5-14	33,704	112	4.5	1.5-10.2	
15-24	30,050	148	6.8	3.3-12.2	
25-44	45,160	273	3.4	1.6-6.3	
45-64	21,035	231	2.6	1.0-5.6	
≥65	12,261	185	2.7	0.9-6.3	
Cultural group					
African/Black	NA	913	4.3	1.7-5.5	0.54
Indian	NA	53	0	NA	
Asian	NA	1	0	NA	
European	NA	2	0	NA	
South American	NA	1	0	NA	
North American	NA	7	0	NA	
Other	NA	10	0	NA	
Monthly household income (EC$)					
0-500	NA	145	4.2	1.5-8.9	0.39
501-1,000	NA	154	4.0	1.5-8.4	
1,001-1,500	NA	109	2.8	0.6-7.8	
1,501-2,000	NA	88	6.8	2.5-14.3	
2,001-2,500	NA	86	7.0	2.6-14.6	
2,500+	NA	169	2.4	0.7-6.0	
Education of mother					
Primary (5-10 years)	43,302	524	3.7	2.2-5.7	0.47
Secondary (11-17 years)	17,515	244	4.6	2.3-8.0	
Certificate/Diploma	4,501	46	6.5	1.4-17.9	
Undergraduate/Graduate	1,989	22	9.5	1.2-30.4	
Postgraduate	-	21	0.0	NA	
Education of father					
Primary (5-10 years)	45,420	364	4.2	2.1-6.8	0.66
Secondary (11-17 years)	13,122	156	5.8	2.7-10.7	
Certificate/Diploma	3,420	38	2.6	0.1-13.8	
Undergraduate/Graduate	2,051	20	10.5	1.3-33.1	
Postgraduate	-	32	3.1	0.1-16.2	

^1^St. Lucia Census 2001;

NA=Not available

**Table 3a.** Prevalence of acute gastroenteritis by gender in BOI study in Saint Lucia

Gender	Residents (N=156,635)	Respondents (n=1,006)	Monthly prevalence of AGI	95% Confidence interval	p value
Male	77,664	405	3.2	1.7-5.5	0.42
Female	78,971	601	4.4	2.9-6.3	
Total	156,635	1,006	-	-	

**Table 3b.** Gender-adjusted prevalence of AGE

Gender	Survey cases			Population (%)	Age-standardized rate (%)
	No. ill	Respondents (No.)	Rate (%)		
Male	13	397	3.27	49.58	1.62
Female	26	592	4.39	50.42	2.21
Total	39	989	-	-	3.84

**Table 4a.** Prevalence of acute gastrointestinal illness (AGI) by age in BOI study in Saint Lucia

Variable	Residents (N=156,635)	Respondents (n=1,006)	Monthly prevalence of AGI	95% Confidence interval
Age (completed years)				
1-4	14,335	8	12.5	0.3-52.7
5-14	-	40	7.5	1.6-20.4
15-24	33,704	112	4.5	1.5-10.2
25-44	30,050	148	6.8	3.3-12.2
45-64	45,160	273	3.4	1.6-6.3
≥65	21,035	231	2.6	1.0-5.6

**Table 4b.** Age-adjusted prevalence of AGE

Age-group (completed years)	Survey cases			Population (%)	Age-standardized rate (%)
	No. ill	Respondents (No.)	Rate (%)		
<1	1	8	12.50	0.62	0.08
1-4	3	40	7.50	8.54	0.64
5-14	5	112	4.46	21.53	0.96
15-24	10	148	6.76	19.20	1.30
25-44	9	273	3.30	28.85	0.95
45-64	6	231	2.60	13.44	0.35
≥65	5	185	2.70	7.83	0.21
Total	39	997	-	-	4.49

### Handwashing and water source

Respondents were asked how often they washed their hands with and without soap before meals and after going to the toilet (Table 6). None of the handwashing practices variable dichotomized into ‘always’ and ‘sometimes/never’ had a significant association with being a case of AGI (before meals with or without soap, p=0.50; before meals with soap, p=0.74; after using the toilet with or without soap, p=0.21; and after using the toilet with soap, p=0.14). Sources and treatment of drinking-water are outlined in Table 7. There was no significant association between drinking-water source and treated water with being a case of AGE.

### Laboratory survey

During the period March 2008–March 2009, 328 samples were received, and 266 diarrhoeal/AGE stool samples were tested for FBD pathogens. Of these, 28 (10.5%) were positive for an FBD pathogen. *Salmonella* was the most common FBD pathogen (53%), followed by *Shigella* (21%). The laboratory practices and data are outlined in Table 8.

**Table 5.** Summary of secondary symptoms experienced by cases of AGE in the population survey, St. Lucia, 2008

Secondary symptom	Number of cases	Percentage of AGE cases
Fever (measured)	4	10
Fever (not measured)	7	18
Blood in stool	4	10
Vomiting	9	23
Abdominal pain	23	59
Headache	9	23
Nausea	8	21
Sore throat	5	13
Cough	7	18
Runny nose	5	13
Sneezing	2	5
Weakness	1	3

**Table 6.** Handwashing practices reported in a population survey, St. Lucia

Handwashing	Frequency	Survey respondents (No.)	Monthly prevalence (%)	95% Confidence interval
Before meals (with or without soap)	Always	594	4.3	2.8-6.2
	Sometimes	380	3.4	1.8-5.8
	Never	16	0	-
Before meals (with soap)	Always	573	4.1	2.6-6.0
	Sometimes	387	3.4	1.8-5.7
	Never	32	6.3	0.8-20.8
After going to the toilet (with or without soap)	Always	805	4.3	3.0-5.9
	Sometimes	180	2.2	0.6-5.6
	Never	10	0	-
After going to the toilet (with soap)	Always	727	4.5	3.1-6.2
	Sometimes	238	2.1	0.7-4.9
	Never	17	5.9	0.1-28.7

**Table 7.** Drinking-water source and treatment practices reported in a population survey, St. Lucia, 2008

Drinking-water source (p=0.47)	Survey respondents	Monthly prevalence (%)	95% Confidence interval
Piped supply	862	3.2	2.6-5.3
Rainwater/cistern	28	0	NA
Bottled water	93	5.6	1.7-11.9
Well	5	0	NA
Filtered	5	20.0	0.5-71.6
Spring	7	0	
Treatment of water before drinking (p=0.50)			
Yes	388	4.4	2.6-7.0
No	571	3.5	2.2-5.4
Treated with: (p=0.55)			
Chlorine	12	10.0	0.3-44.5
Boiling	229	4.0	1.8-7.4
Filter	153	4.6	1.9-9.3
Sunlight	1	0	NA

NA=Not available

### Estimation of underreporting of AGE to the national surveillance

**Figure 2.**
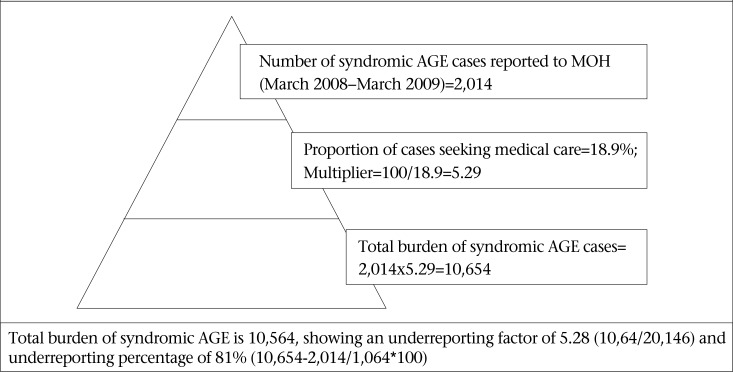
Estimation of underreporting and burden of syndromic acute gastrointestinal illness

**Figure 3.**
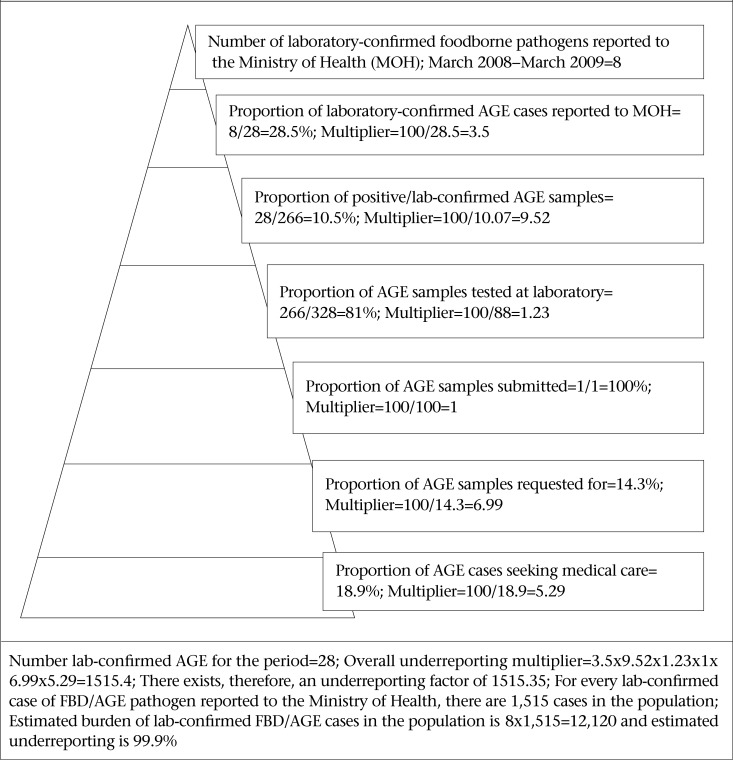
Estimation of underreporting and burden of laboratory-confirmed foodborne pathogens

Table 8 defines the information and variables used in constructing the surveillance pyramids seen in Figure 2 and 3 and to calculate the burden of AGE for syndromic AGE and laboratory-confirmed FBD. Figure 2 and 3 show the surveillance pyramids defined and the information to calculate the burden of AGE for syndromic AGE and laboratory-confirmed FBD. Using data of the syndromic surveillance of AGE, the estimated burden of AGE for one year period from March 2008 to March 2009 in St. Lucia was 10,675. The number of syndromic AGE cases reported to the Ministry of Health for the same period was 2,040. Thus, there was an underreporting factor of 5.28 (10,64/20,146) and underreporting percentage of 81% (10,654-2,014/1,064*100) for syndromic AGE (Figure 3). For every 1 case of AGE reported to the Ministry of Health, 5.28 more cases were occurring in the population. Using the laboratory surveillance data, the estimated burden of laboratory-confirmed AGE for the one year period from March 2008 to March 2009 was 12,120. There is an underreporting factor of 1,515 and estimated underreporting percentage of 99.9% for laboratory-confirmed foodborne/AGE pathogens in St. Lucia (Figure 3).

**Table 8.** Information required for the calculation of burden of AGE in St. Lucia, March 2008–March 2009

Information required	Source	Value
Total no. of AGE reported to the Ministry of Health (March 2008–Mach 2009)	Ministry of Health, Surveillance Unit	2,040
Laboratory cases reported tothe Ministry of Health (March 2008–March 2009)	Surveillance Unit	8/28=28.5%
Number of FBD pathogens isolated at the laboratory	Laboratory	28
How sensitive is the laboratory tests?	Laboratory	95–98%
Proportion of tests positive for an FBD pathogen	Laboratory	28/266=0.5%
Specimen submitted: How often were stools specimens tested?	Laboratory	% Total AGI specimens submitted/total number tested (266/328)=68.2%
How often were stools specimens submitted?	Population survey	100%
How often were stools specimens requested for?	Population survey	14.3%
How often did ill persons seek medical care? (n=7)	Population survey	18.9%

### Socioeconomic costs

The estimated cost of treating an AGE case in St. Lucia was calculated to be EC$ 987 (US$ 365) (Appendix 2), including cost of medication. Using this estimate, the annual economic burden on patients associated with treatment of syndromic AGE was estimated to be US$ 3,892.837.36

## DISCUSSION

Saint Lucia was the first country to initiate the Burden of Illness (BOI) Study in the Caribbean as part of the overall regional Caribbean BOI study being coordinated by CAREC/PAHO/WHO during 2007-2012. It was a pilot study and, in addition to determining the burden of AGE-related illness and its economic impact in St. Lucia, the lessons learnt from implementing this pilot study were used in improving the implementation of BOI studies in the other Caribbean countries (Grenada, Guyana, Trinidad and Tobago, Jamaica, Dominica, Barbados, Bermuda, and Belize). The estimated economic costs of acute gastroenteritis (AGE) in St. Lucia (US$ 365/case and US$ 3,892.837.36/year) provide strong evidence to the policy-makers and the Ministry of Finance to persuade the allocation of limited resources in a developing country, like St. Lucia, to AGE and FBD surveillance, prevention, and control.

Burden of illness studies have not been conducted previously in Saint Lucia. This study provided evidence to demonstrate that acute gastrointestinal illness and foodborne diseases represent a substantial health and economic burden on the developing nation of St. Lucia. It has also shown a major gap of underreporting of both syndromic AGE and laboratory-confirmed FBD data to the Ministry of Health. This information will assist St. Lucia to determine appropriate prevention and control measures for FBD, identify gaps in surveillance and target the identi­fied risk factors to guide the allocation of resources for education, food safety, and infrastructure, which will lower the morbidity associated with acute gastrointestinal illness. Our data will also be collated with data from the other countries to determine the burden of AGE and FBD illness in the Caribbean. This will not only provide information to guide regional interventions and policies but will also inform the WHO's BOI initiative in determining the global burden of FBD.

Acute gastrointestinal illness is an important public-health issue worldwide. Several studies on gastrointestinal disease of similar design have been conducted in large populations and mostly in developed countries (1,3). This pilot study was in the developing country-setting of St. Lucia where the surveillance system for AGE is not as developed as in some other developing countries, and it charted the way forward for the Caribbean region. The logistics in the execution of this study were pivotal in obtaining reliable and useful outcomes. Through this pilot study, we learned that (i) a survey coordinator with specific responsibilities was essential for improving the overall implementation of the study, quality of data collection, and speed of processing; following this, a BOI coordinator was identified in all the other subsequent studies; (ii) conducting interviews during the day-time (8:30 am-4:30 pm) led to the selection of the convenient selection of females (housewives who were at home); subsequently, the timing of interviews was changed to evenings and weekends in the other countries to ensure that we selected the right respondent in the household (i.e. person with the next birthday ahead); (iii) it is necessary that all replacement interviewers be properly trained in conducting face-to-face interviews and retraining of interviewers before the second surveys in ach country; (iv) it is essential that all interviewers be familiar with the area, landscape, and household maps of the areas that they were conducting the interviews in; (v) daily monitoring of completed interviews should be continued to ensure that they were being done correctly; (vi) it is imperative that interviewers be familiar with the terrain of some of the rough/hard-to-reach and/or rural areas (characteristic of Caribbean countries) and that they were always accompanied by local guides in these hard-to-reach rural areas; and (vii) the importance of the added value of using enumerators/persons who have experienced in conducting population survey opposed to those who lack experience.

The 3.9% monthly prevalence of AGE and incidence of 0.5 episodes per person-year found in our study were considerably lower than that found in other countries. BOI studies in Cuba, Chile, and Canada showed AGE prevalence of 10.6%, 9.2%, and 9.2% respectively while studies in Norway, Canada, and the USA had AGE annual incidence rates of 1.2, 1.3, and 1.4 episodes per person-year respectively (4-6,8-11) However, a study done in Northern Ireland and the Republic of Ireland showed an incidence of 0.6 per person-year (7). However, despite this lower prevalence, the estimated burden and underreporting of syndromic AGE (10,674 cases for the study period, 81%) and laboratory-confirmed AGE/ FBD pathogens (12,012 cases, 99.9%), coupled with the estimated economic burden of almost US$ 3,892.837.36 (US$ 365/case) indicate that AGE and FBD-related illness pose a considerable health, economic and social burden on St. Lucia. Underreporting of illness is a major problem for both clinical management and public health interventions. The high underreporting (81% for syndromic AGE and 99.9% for laboratory-confirmed FBD pathogens) identified in this study reflects poor coordination of surveillance between the national laboratory and the National Surveillance Unit as well as poor collection of syndromic data from the health regions in St. Lucia. Thus, immediate measures should be put in place to improve the surveillance (from data collection to reporting, both syndromic and laboratory-based) and prevention of AGE and FBD illnesses to avoid the high health and economic burden of this illness on the population of St. Lucia.

Our finding of the high burden of AGE has grave implications for the tourism industry in St. Lucia, this being a tourism-dependent country. Travellers’ diarrhoea is common among visitors to the Caribbean and can result in significant negative impact, including decline in arrivals, loss of reputation, negative publicity, lawsuits, hotel closures and, hence, the sustainability of the tourism industry and the economy (19). It is imperative that St. Lucia offers a safe and healthful destination and put measures in place to reduce AGE and FBD-related illness, and improve food safety for all visitors. Our study did not assess the burden of AGE among visitors to St. Lucia; however, further research is definitely needed in this area.

Our finding of a higher prevalence of AGE in children aged less than 5 years is similar to many studies (3-11). Age is a known risk factor for AGE, FBD, and other communicable diseases. Poorer hygiene in the very young, opposed to older groups, could also be a contributing factor to this finding. The secondary symptoms identified from the AGE cases were similar to that noted in other AGE studies (7-11).

The low response rate in the first population survey (Phase 1) (79%) compared to second survey (Phase 2) (98%) may be due to the use of smaller numbers of interviewers used in the first phase (20-25 persons), requiring a higher number of surveys to be done by an interviewer and fact that the nurse interviewers conducted this survey during their working hours (8:30 am-4:30 pm), in addition to other duties. When these factors were changed in the second population survey to include a higher number of interviewers (40-50 interviewers, averaging 3-5 surveys/interviewer/day) and conducting the interviews was made the primary task of the interviewers as well as the retraining of all interviewers and replacements, a much higher response rate was obtained (98%). In addition, interviewers were assigned according to the enumeration district they lived in, and a study coordinator was identified in the second phase, whose sole role was to ensure the timely and appropriate administration of the survey, which is important for greater success. In the second phase, response rates were also higher due to a greater commitment to reaching the selected respondents at whatever time of the day, night, or weekends they were available. Enumerators were also chosen as interviewers opposed to community nurses, the former having more experience in conducting surveys as well as tested knowledge and ability to follow household maps in rugged terrain.

Females, persons aged 25-44 years, and income level were overrepresented in the survey compared to the census population. The apparent bias in the selection of interviewees was noted, leading to overrepresentation of females and persons aged >25-44 and ≥65 years and underrepresentation of children aged <10 years; these suggest that some interviewers chose a ‘convenient’ respondent, i.e. the person who was at home during the time rather than the person with the next birthday ahead, which was the methodology used for randomization in the household. In St. Lucia, the survey was conducted by nurses during 8:30 am-4:30 pm (working hours) and since more females (likely to be unemployed housewives) tend to be at home during the day than males, children, and older people, this may have led to the overrepresentation of females over persons in the 25-44 and ≥65 years age-group in this study. These differences from general population demonstrate a selection bias in the sample and weaken the results of the study. It represents a limitation in our study and suggest that there is the need for enhanced and daily monitoring of interviewers’ practices and more stringent training with interviewers, emphasizing the need and benefit of randomization and to interview the right person to ensure more representativeness of the data in the future surveys in these countries as well as in others. Replacement interviewers should also be retrained.

Our study indicated a low tendency to seek medical care for AGE-related illness (18%). This was similar to other countries since people with AGE/diarrhoeal illness tend to self-treat rather than seeking medical care. In fact, medical attention is generally sought when kids are involved or when the illness is severe. This finding may also reflect poor expectations for medical attention in St. Lucia and the wholesomeness and reliance on the medical system.

The finding that 18% of the cases reported other individuals in their home having AGE at the time of their AGE-related illness indicated that family outbreaks of AGE were occurring However, these outbreaks/clusters were not reported/detected by the national surveillance system, suggesting a gap in the system. Furthermore, the finding that only one stool was requested for emphasizes the well-known fact that stool collection from AGE cases is not a common practice in the Caribbean. This is a major gap in the surveillance of AGE in St. Lucia, and it severely limits the determination of aetiology of AGE and FBD-related illness in the country. Laboratory isolation of the pathogens from stools is essential for determining causation of illness, medical management, and the development of appropriate prevention measures. We recommend that stool collection be enhanced and the range of FBD pathogens tested for be increased to improve the surveillance of AGE and FBD-related illnesses in St. Lucia.

Finally, our study noted a high incidence of loss of activities (76.9%) in persons who were affected with AGE and that 8% of persons who were sick stayed at home for over 2 days, on average, either from work or school. This speaks of the significant economic burden of AGE-related illness and will impact on the country both socially and economically and further contribute to the economic burden of AGE-related illness.

### Conclusions

Saint Lucia was the first country to conduct a burden of illness (BOI) study in the Caribbean. The study has demonstrated that AGE and FBD-related illness pose a significant health and economic burden on the population of St. Lucia and that immediate measures should be taken to improve the surveillance of AGE and FBD-related illness, reduce its underreporting to the Ministry of Health, and improve food safety practices. This would contribute to reducing the burden of AGE and FBD in St. Lucia and making the island safer and healthier for both its local and tourist population.

## ACKNOWLEDGEMENTS

We thank the Caribbean Epidemiology Centre (CAREC) and the Pan American Health Organization (PAHO) for their pivotal roles in providing financial and technical assistance. We express our immense gratitude to the Public Health Agency of Canada (PHAC) for their unwavering support throughout this project, with special mention of James Flint, Andrea Currie, and Kate Thomas. We also thank the Caribbean Eco-Health Programme and the Global Health Research Initiative for assisting with funding. Special mention and thanks to the former Minister of Health of Saint Lucia Dr. Keith Mondesir, the former Permanent Secretary Mr. Felix St. Hill and Chief Medical Officer Dr. Josiah Rambally. We thank the Ethics Committee and the staff of the Epidemiology Unit in the Ministry of Health, with special mention of Ms Crissah Emmanuel and Mr. Alvin Caesar. This project could not have been completed without the input of Principal Nursing Officer (PNO) Mrs. Ann Margaret Henry and Assistant PNO Mrs. Juliette Joseph. They were instrumental in coordinating the efforts of the public health nurses throughout the island.

## Figures and Tables

**Appendix 1.**
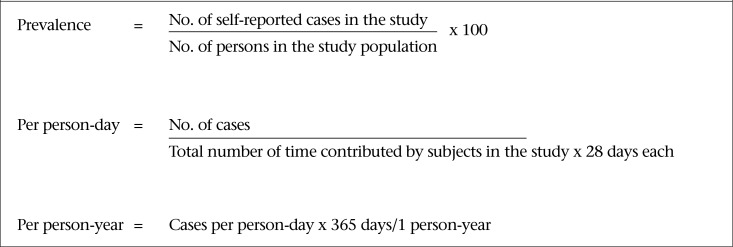
Formulae used in calculating prevalence and incidence rate per person-year in the burden of acute gastrointestinal illness study in Saint Lucia

## Appendix

**Appendix 2.** Calculation of costs of burden

Category of cost		Private healthcare (EC$)	Public healthcare (EC$)	Average (EC$)
Medical visit		150.00	20.00	85.00
Medication	- Analgaesic/Antipyretic	20.00	5.00	12.50
	- Oral rehydration salts (ORS)	10.00	5.00	7.50
	- Antibiotics	40.00	15.00	27.50
	- Gravol	18.00	8.75	13.38
	- IV	325.00	175.00	250.00
Lost work (3 days) (Middle income)		460.00	275.00	367.50
Caregiver		300.00	150.00	225.00
Total		1,323.00	653.75	987.50

1 EC$=0.3702 US$
